# Harnessing optical bound states in the continuum for ultrafast, reconfigurable, long-range photonic networks

**DOI:** 10.1038/s41377-025-02071-x

**Published:** 2026-01-04

**Authors:** Jiantao Ma, Ying Yu, Jin Liu

**Affiliations:** State Key Laboratory of Optoelectronic Materials and Technologies, School of Electronics and Information Technology, School of Physics, Sun Yat-sen University, Guangzhou, 510275 China

**Keywords:** Photonic devices, Micro-optics

## Abstract

Bound states in the continuum (BICs) provide a route to strong, long-range photonic coupling with dynamic tunability. Recent advances demonstrate that BIC metasurfaces enable reconfigurable two-dimensional coupling between arbitrarily positioned resonators, with the added capability of ultrafast all-optical control.

Large-scale, reconfigurable photonic circuits are a central goal for optical computing^1,2^ and quantum technologies^3,4^. While individual micro- or nanophotonic devices have achieved outstanding performance^5–7^, scaling up to large-scale networks is limited by the intrinsically short-range nature of conventional (evanescent) coupling: tight field confinement that benefits an individual resonator also confines intercavity interactions to within a fraction of a wavelength^8–10^. Attempts to extend range—using epsilon-near-zero media, hyperbolic metamaterials, or related engineered platforms—often face a range–strength trade-off and tight design constraints^11–14^. Waveguide buses carry light over long distances but effectively impose one-dimensional interconnects and strict spectral matching, thereby complicating reconfigurability and scale-out^15,16^.

Against this backdrop, optical bound states in the continuum (BICs)^17,18^—non-radiating modes embedded in the continuum—offer a distinct approach. BIC platforms support ultra-high-Q resonances^19,20^, admit momentum-space topology control^21–23^, and have enabled low-threshold microlasers^24,25^, yet are often associated with large periodic structures. In a recent study published in *Light: Science & Applications*, a team led by Prof. Qinghai Song at the Harbin Institute of Technology (Shenzhen, China), turns this feature into an advantage for networking, using a metasurface-supported BIC as a shared, non-radiating channel to mediate long-range, reconfigurable coupling among optically written quasi-BIC microlasers on a single chip^26^—serving as a remarkable breakthrough toward future photonic networks.

As illustrated in Fig. 1a, Tang et al. demonstrate that focusing pump spots at arbitrary positions on a BIC-supporting metasurface writes quasi-BIC microlaser nodes without lithographic changes^26^. Under finite-size pumping, the otherwise delocalized BIC locally converts to a high-Q quasi-BIC—due to spatial confinement^23^—yielding lasing in the illuminated regions. Because the non-radiative BIC mode extends across the metasurface, spatially separated nodes become inherently coupled through this shared channel, bypassing the usual range–strength compromise. All nodes lase at the same wavelength set by the lattice, ensuring spectral uniformity across the network. As a result, two-dimensional coupling graphs are defined by pump patterns rather than fixed routing, and interaction distances extend from the near field to tens of micrometers, with access to non-Hermitian laser physics (e.g., zero-mode lasing) on the same platform^26^.

**Fig. 1:**
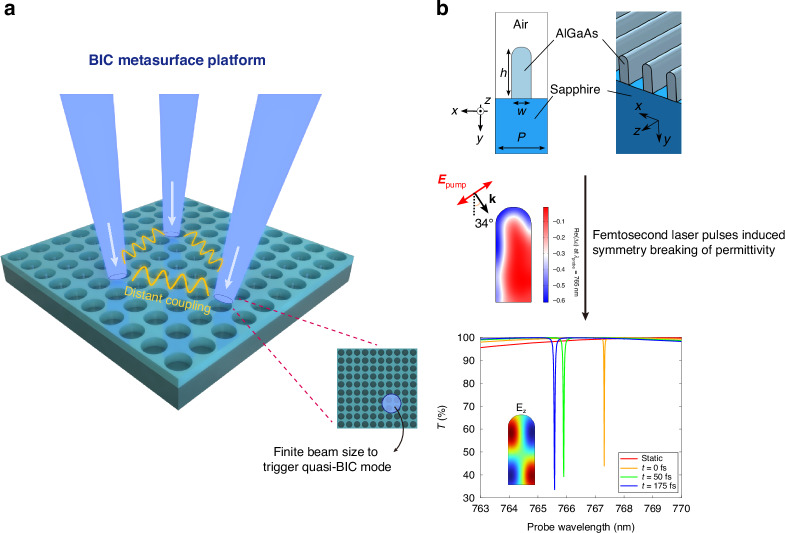
Schematics of a BIC metasurface platform for ultrafast, reconfigurable, long-range photonic coupling. **a** Interactions between quasi-BIC microlasers. Quasi-BIC state is generated from an ideal BIC state under finite-size pumping. Inset: pump beam profiles on metasurface. Images reproduced from ref. ^26^. **b** A complementary strategy for ultrafast dynamic BIC manipulation. Top: Cross-section of the unit cell of the 1D-periodic nanowires. Middle: Asymmetric permittivity distribution on the unit cell induced by femtosecond laser pulses. Bottom: Transmission spectra of the 1D periodic nanowire metasurface at different time delays after the pump peak, indicating the switching-on of quasi-BIC states. Images reproduced from ref. ^27^

Complementing this active spatial programmability is a separate study by Giulia Crotti et al. from Politecnico di Milano, published in *Light: Science & Applications*, which introduces a temporal control mechanism for BIC-derived resonances via transient optical symmetry breaking^27^ (Fig. 1b). Their theoretical proposal shows that femtosecond-pulse excitation of a symmetric semiconductor metasurface can induce a spatially inhomogeneous hot-carrier distribution, breaking the in-plane symmetry protecting the BIC and thereby switching on a quasi-BIC resonance on sub-picosecond timescales. Because symmetry restoration through carrier diffusion occurs much faster than full carrier recombination, the resonance can be toggled at speeds beyond conventional thermo-optic or free-carrier modulation. Importantly, this approach does not require pre-fabricated geometric asymmetry^19,20,28^. Viewed together with the results of Tang et al., these studies provide orthogonal control axes—spatial/gain programming for network topology and ultrafast gating for state selection—within a unified BIC framework^26,27^.

Looking ahead, BIC-mediated spatiotemporal programmability may support a software-defined optical backplane. For photonic and AI computing, a metasurface offering long-range, spectrally uniform coupling among optically addressable nodes could serve as a reconfigurable weight fabric for matrix operations, while femtosecond-scale symmetry-breaking and gain timing might enable on-the-fly updates to weights and connectivity without lithography^29–31^. In parallel, combining BIC backplanes with topological photonics may offer long-reach yet disorder-tolerant transport, and operation in non-Hermitian regimes can be leveraged for sensitivity enhancement in programmable sensing^32^. Beyond computing, quantum photonics may benefit from BIC channels as low-loss buses for deterministic coupling between distant emitters^10,33,34^. Realizing these possibilities will likely require co-design across materials, control electronics, and algorithm–hardware stacks, together with inverse-designed and machine-learning-assisted photonic components that use BIC topology as a design prior^35^. With continued progress, BIC platforms appear well positioned to connect single-resonator excellence to ultrafast, reconfigurable, long-range photonic systems at scale.
